# Helicopter-borne RGB orthomosaics and photogrammetric digital elevation models from the MOSAiC Expedition

**DOI:** 10.1038/s41597-023-02318-5

**Published:** 2023-07-03

**Authors:** Niklas Neckel, Niels Fuchs, Gerit Birnbaum, Nils Hutter, Arttu Jutila, Lena Buth, Luisa von Albedyll, Robert Ricker, Christian Haas

**Affiliations:** 1 Alfred Wegener Institute, Helmholtz Centre for Polar and Marine Research, Bremerhaven, 27570 Germany; 2 Institute of Oceanography, Center for Earth System Research and Sustainability (CEN), Universität Hamburg, Hamburg, Germany; 3 Cooperative Institute for Climate, Ocean and Ecosystem Studies, University of Washington, Seattle, WA 98105 USA; 4 NORCE Norwegian Research Centre, Tromsø, 9019 Norway; 5 Institute of Environmental Physics, University of Bremen, Bremen, 28334 Germany; 6 Present Address: Finnish Meteorological Institute, Helsinki, 00560 Finland

**Keywords:** Cryospheric science, Physical oceanography, Imaging and sensing

## Abstract

The Multidisciplinary Drifting Observatory for the Study of Arctic Climate (MOSAiC) expedition took place between October 2019 and September 2020 giving the rare opportunity to monitor sea-ice properties over a full annual cycle. Here we present 24 high-resolution orthomosaics and 14 photogrammetric digital elevation models of the sea-ice surface around the icebreaker RV *Polarstern* between March and September 2020. The dataset is based on >34.000 images acquired by a helicopter-borne optical camera system with survey flights covering areas between 1.8 and 96.5 km^2^ around the vessel. Depending on the flight pattern and altitude of the helicopter, ground resolutions of the orthomosaics range between 0.03 and 0.5 m. By combining the photogrammetric products with contemporaneously acquired airborne laser scanner reflectance measurements selected orthomosaics could be corrected for cloud shadows which facilitates their usage for sea-ice and melt pond classification algorithms. The presented dataset is a valuable data source for the interdisciplinary MOSAiC community building a temporal and spatially resolved baseline to accompany various remote sensing and *in situ* research projects.

## Background & Summary

Between October 2019 and September 2020 the icebreaker RV *Polarstern*^1^ served as the logistical center of the *Multidisciplinary drifting Observatory for the Study of Arctic Climate* (MOSAiC) expedition. While drifting through the central Arctic, the main goal of the MOSAiC expedition was to collect various datasets related to the disciplines atmosphere, sea ice, ocean, ecosystem and biogeochemical processes over a full annual cycle^2^. The dataset introduced here is likely of value to the wider MOSAiC community as it provides the spatio-temporal change of the sea ice (or snow) surface conditions during the drift experiment. In particular, we present high resolution (sub-meter scale) orthomosaics and photogrammetrically derived Digital Elevation Models (DEMs) of the ice floes surrounding RV *Polarstern* on her drift across the Arctic Ocean. These are especially relevant but not restricted to studies on meltpond evolution, melpond drainage and volume, albedo changes, floe size distribution, and the validation of lower resolved satellite retrievals.

Our dataset is build upon >34.000 RGB camera images acquired on helicopter flights between March and September 2020 as part of the weekly schedule of the MOSAiC snow and sea ice team. The camera imagery is accompanied by position data of the helicopter and contemporaneously acquired Airborne Laser Scanner (ALS) data. The core of our analysis is a Structure from Motion (SfM) approach which uses the principle of motion parallax to reconstruct three-dimensional surfaces of the surveyed area. The derived surface models are then textured with brightness-adjusted camera imagery to generate seamless orthomosaics for each survey. Today this technique is widely used in various disciplines of environmental science, engineering, and archaeology to obtain high-resolution three-dimensional mapping results at reasonable costs^3–7^. SfM was also applied to imagery from airborne sea-ice surveys acquired by helicopters or drones^8,9^. When compared to land surveys sea ice moves considerably within the survey’s time window requiring an initial drift correction of the image locations before applying SfM. For this, we employed the position information of RV *Polarstern* at the time of image acquisition. However, a lack of Ground Control Points (GCPs) on the moving ice floes introduces certain tilts and biases in the derived data products. We, therefore, rely on additional gridded ALS surface elevation data that we employed as pseudo ground control data to align the photogrammetric models. Next to the ALS surface models we also used gridded laser reflectance values, which are of great value to correct the derived orthomosaics for the effects of cloud shadows.

This study is organized in the following way. At first we give a brief overview of the data acquisition setup and the locations and times of the helicopter surveys. Then the conversion and brightness adjustment of the RAW camera images is described, followed by a detailed description of the SfM workflow. Next, we describe the combination of the derived camera products and the ALS data and show the benefits of simultaneous data acquisition of both instruments. Finally, we evaluate the impact of the applied drift correction and compare the results with contemporaneously acquired satellite data (i.e. <7 h time difference to the respective helicopter survey) and independent airborne surface temperature measurements.

## Methods

### Data acquisition setup

**Fig. 1:**
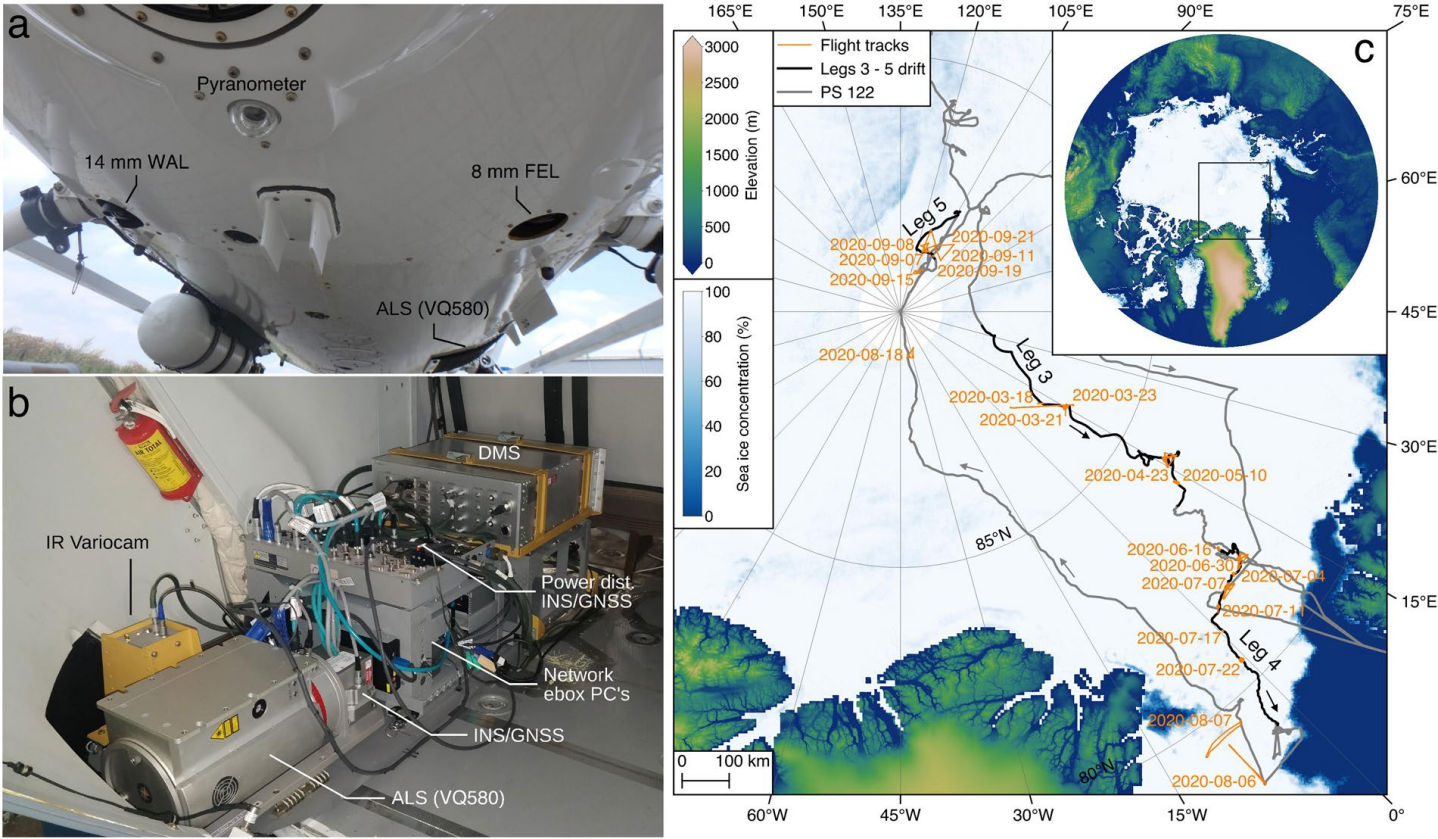
Overview of helicopter setup and RGB camera surveys conducted during the MOSAiC expedition. Panel **a** shows the helicopter’s bottom face including the position of the 14 mm Wide Angle Lens (WAL) used in this study (picture provided by Christian Royal). The instrumentation in the cargo compartment of the helicopter is shown in panel **b** and includes among others the employed INS/GNSS unit and the Airborne Laserscanner (ALS). Note that the IR Variocam was only flown until the end of leg 3 (picture provided by Stefan Hendricks). Panel **c** shows the drift/cruise of RV *Polarstern* during the MOSAiC campaign (PS122) with the locations of the helicopter flights indicated in orange. Surface elevations are from the ALOS World 3D - 30 m (AW3D30) dataset^39,40^ overlayed with sea-ice concentration on 2020-05-31^41^.

In this study, we employed RGB camera imagery acquired by a CANON EOS-1D Mark III Digital Single Lens Reflex camera, which incorporates a Complementary Metal Oxide Semiconductor image sensor providing three channels for RGB acquisitions capturing a wavelength range between 400 nm and 700 nm (AWI SENSOR ID: 4430, RGBCAM_WAL_HELI). The camera was deployed with a CANON 14 mm f/2.8 L II Ultra Sonic Motor lens and mounted as nadir looking imaging system aboard a Eurocopter BK 117 C-1 (Fig. 1a). All camera data were recorded in uncompressed and unprocessed RAW data format. Data obtained at microseconds precision with an Applanix AP60 AV combined Inertial Navigation System (INS) and Global Navigation Satellite System (GNSS) were used to provide geographical position and image orientation for the aerial imagery. The GNSS/INS system was installed in combination with a Riegl VQ580 Airborne Laser Scanner (ALS) in the cargo compartment of the helicopter (Fig. 1b). In order to obtain synchronized time information a handheld GARMIN GPS 60Csx was connected to the camera and its UTC time tag with seconds precision was directly written into the EXIF metadata of the camera imagery. During the survey flights images were taken at a frequency of 0.25 Hz at an average flight speed of ~150 km/h (Table 1).

**Table 1:** List of evaluated helicopter surveys conducted during legs 3-5 of the MOSAiC expedition.

Date	#Flight	DSHIP^19^ ID	Altitude (m)	t_*start*_	t_*center*_	t_*stop*_	Δ t	Distance (km)	Flight pattern
2020-03-18	1	PS122/3_32-42	319.0	10:48:00	10:52:20	10:56:36	0:08:36	23.4	CO floe grid
2020-03-21	1	PS122/3_32-70	329.4	08:10:52	09:09:16	10:37:28	2:26:36	344.3	CO floe grid
2020-03-23	1	PS122/3_33-17	297.7	10:40:46	11:28:52	12:17:00	1:36:14	262.0	Transect
2020-04-23	1	PS122/3_37-63	331.0	07:44:18	08:38:06	09:32:02	1:47:44	287.9	CO floe grid
2020-04-23	2	PS122/3_37-66	328.6	10:05:10	10:46:54	11:27:10	1:22:00	220.7	L-site triangle
2020-05-10	1	PS122/3_39-109	329.1	13:57:28	14:53:18	15:43:58	1:46:30	284.8	CO floe grid
2020-06-16	1	PS122/4_44-78	193.1	09:22:06	10:04:42	10:47:14	1:25:08	205.0	CO floe grid
2020-06-30	1	PS122/4_45-36	341.9	07:39:30	08:23:42	09:08:38	1:29:08	223.5	CO floe grid
2020-06-30	2	PS122/4_45-37	339.6	09:33:37	10:16:29	11:01:37	1:28:00	221.2	L-site triangle
2020-07-04	1	PS122/4_45-112	104.7	08:47:45	09:44:09	10:32:05	1:44:20	112.1	CO floe grid
2020-07-07	1	PS122/4_46-36	101.2	08:57:49	09:57:29	10:57:05	1:59:16	109.7	CO floe grid
2020-07-07	3	PS122/4_46-39	153.0	12:28:01	13:27:29	14:28:57	2:00:56	219.2	L-site triangle
2020-07-11	1	PS122/4_46-97	173.1	11:20:31	11:44:38	12:08:42	0:48:11	94.5	Transect
2020-07-17	1	PS122/4_47-96	105.7	15:22:49	16:14:13	17:08:37	1:45:48	58.8	CO floe grid
2020-07-22	1	PS122/4_48-69	336.7	15:15:10	16:21:06	17:28:14	2:13:04	301.2	CO floe grid
2020-08-06	1	PS122/4_50-32	264.9	08:53:18	09:48:50	10:44:14	1:50:56	238.6	Transect
2020-08-07	1	PS122/4_50-45	253.9	08:35:01	09:26:25	10:17:53	1:42:52	224.6	Transect
2020-08-18	2	PS122/5_59-139	321.9	15:33:09	15:57:16	16:21:24	0:48:15	106.1	Transect
2020-09-07	1	PS122/5_61-62	78.7	04:21:33	05:19:29	06:17:13	1:55:40	85.3	CO floe grid
2020-09-08	2	PS122/5_61-63	311.1	12:23:01	13:07:17	13:51:37	1:28:36	188.8	Butterfly
2020-09-11	1	PS122/5_61-190	77.4	05:32:48	06:37:24	07:41:56	2:09:08	88.1	CO floe grid
2020-09-15	1	PS122/5_62-67	82.5	04:44:07	05:31:19	06:20:24	1:36:17	71.9	CO floe grid
2020-09-19	1	PS122/5_62-166	300.9	10:03:30	11:05:34	12:02:34	1:59:04	293.8	CO floe grid
2020-09-21	1	PS122/5_63-3	233.3	07:45:51	08:41:51	09:37:47	1:51:56	240.0	Butterfly

Next to the date, flight number and DSHIP^19^ (AWI’s Data Acquisition and Management System for technical, nautical, and scientific data) ID, we list the average flight altitude, the acquisition times of the first (*t*_*start*_), central (*t*_*center*_) and last image (*t*_*stop*_), the duration of the image recording period (Δ*t*), the flight distance covered and the flight pattern of the survey flight. The latter is sorted into Central Observatory (CO) grid flights, Transect flights, L-site triangle flights, and Butterfly flights. All images were drift corrected to time *t*_*center*_ of the respective flight. All time values are given in Coordinated Universal Time (UTC) format indicated as HH:MM:SS.

### Data overview

**Fig. 2:**
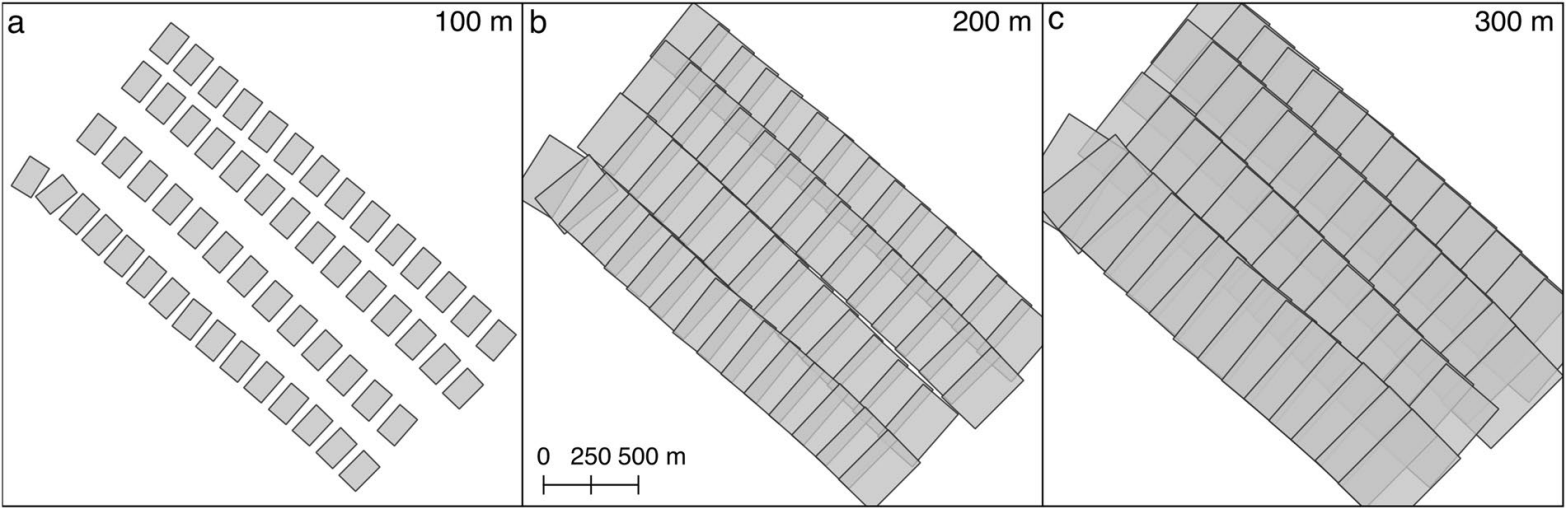
Relation between flight altitude and image overlap. Image footprints for simulated flight altitudes of 100 m, 200 m, and 300 m are shown employing extrinsic and intrinsic camera information from the MOSAiC camera setup.

Between October 2019 and September 2020 the MOSAiC expedition was conducted within 5 legs with RV *Polarstern* moored to three different ice floes called Central Observatories (CO)^2^. During all legs, helicopter surveys were flown in the wider vicinity of RV *Polarstern* to monitor the temporal evolution of the snow and sea-ice conditions during the drift. Using the data acquisition setup described above four different flight patterns were conducted: (1) floe grid flights covering the CO and its neighboring ice floes (2) L-site triangle flights connecting a 3 buoy pattern (3) butterfly surveys connecting a 4 buoy pattern with RV *Polarstern* in the center and (4) transect flights for various scientific and logistical reasons. To obtain robust results from the SfM pipeline certain image requirements must be fulfilled. These include (1) solar illumination (2) no low clouds or fog and (3) sufficient image overlap. We therefore only employed data acquired during legs 3–5 (March to September 2020, Fig. 1c). Furthermore, we focused on data acquired within grid flights or at high-altitude line flights. For the latter, it turns out that the standard configuration described above requires an altitude of >300 m to obtain appropriate image overlap along-track. Line flights conducted at lower altitudes only have results in areas of overlapping flight tracks. This also becomes evident when looking at Fig. 2 where we simulated the image overlap on the sea-ice surface using extrinsic and intrinsic camera information from the MOSAiC camera setup and different flight altitudes of 100 m, 200 m, and 300 m. All survey flights evaluated in this study are listed in Table 1 with their geographic position indicated in Fig. 1c.

### Production of RGB orthomosaics and photogrammetric DEMs

**Fig. 3:**
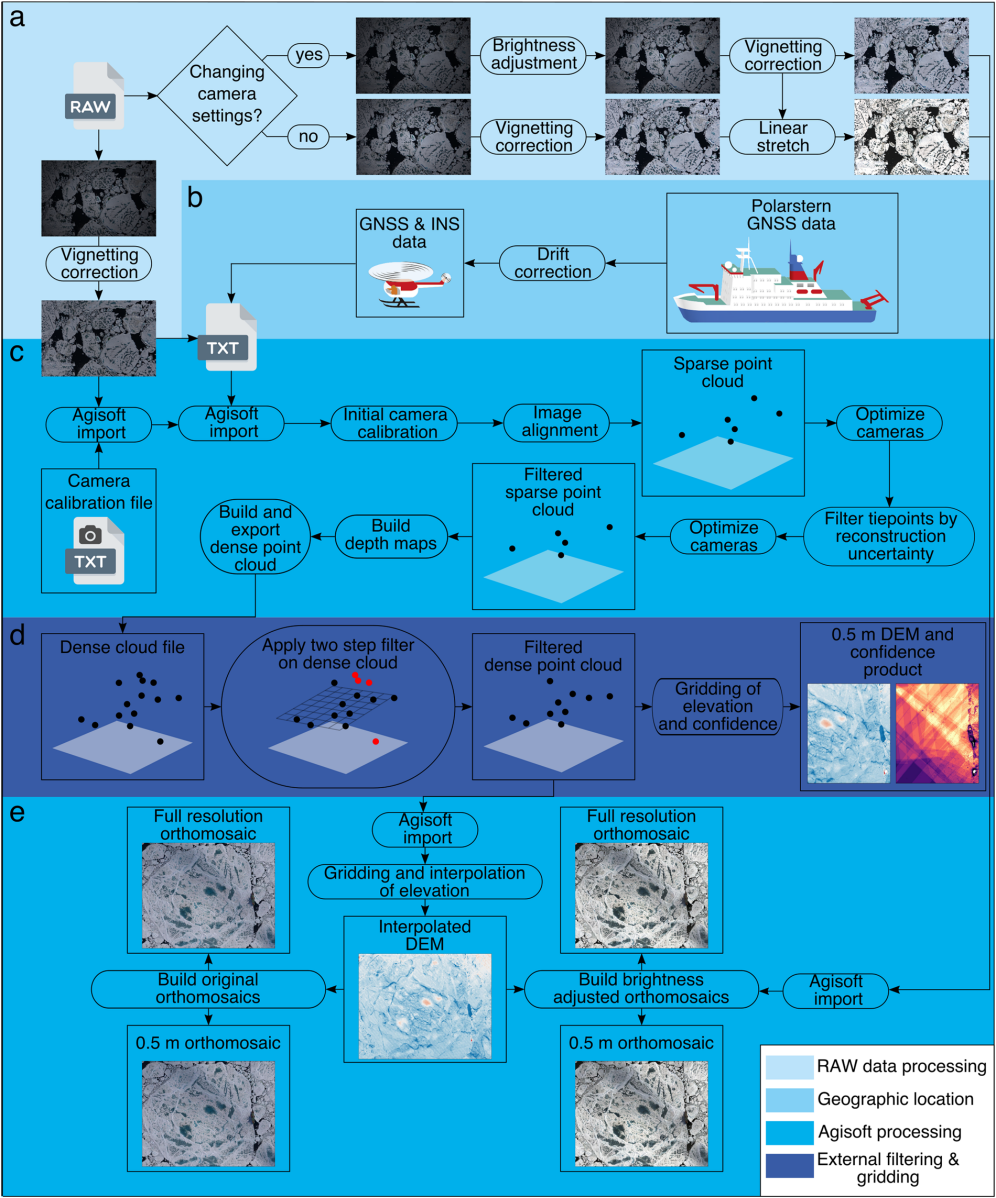
Flowchart in the production of RGB orthomosaics from helicopter-borne camera surveys conducted during the MOSAiC expedition.

In this section we describe the photogrammetric production of orthomosaics and DEMs from the RGB camera data acquired during the survey flights listed in Table 1. Figure 3 summarizes the methods applied and the single processing steps are addressed in more detail below.

#### Image conversion, vignetting and initial brightness correction

In the first step RAW images were converted to Tagged Image File Format (TIFF) employing the dcraw tool^10^ with different brightness corrections for each flight. In order to achieve a continuous image brightness within each flight all images were adjusted with respect to the applied camera settings if these were changed during the survey (Fig. 3a). Brightness-relevant camera settings are Aperture (F-number), Shutter Speed and ISO value. These three variables are included in exiftool’s^11^ Light Value (*LV*) which is defined by

1 $$LV=2\times lo{g}_{2}\left(Aperture\right)-lo{g}_{2}\left(ShutterSpeed\right)-{{\rm{\log }}}_{2}\left(\frac{ISO}{100}\right).$$

**Fig. 4:**
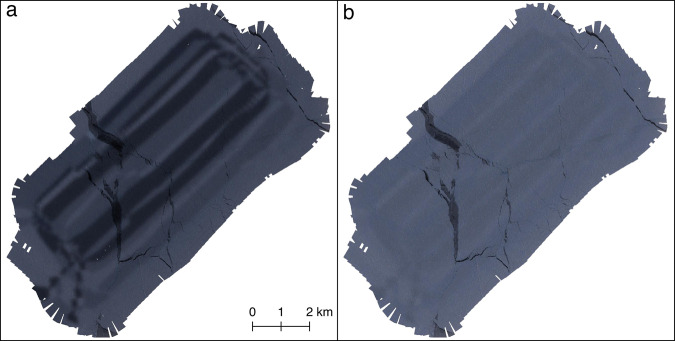
Orthomosaic of survey flight 20200321_01_PS122-3_32-70. Panel **a** shows the results of the original imagery with varying brightness settings during the flight, while panel **b** shows the same data after applying the brightness correction scheme described in the text.

When exposure settings varied during one flight, we calculated a brightness correction factor for each image. The lowest *LV* of the associated flight (i.e. the brightest image of the flight) was successively subtracted from the single *LV*’s of the flight. The resulting brightness correction factors were then applied during the RAW to TIFF conversion of the images. An example is shown in Fig. 4 where different brightness settings were applied during a survey flight. Figure 4a shows the final orthomosaic without the proposed brightness correction while Fig. 4b shows the results after applying the brightness correction scheme described above. Here it should be noted that for the majority of the flights all brightness settings were kept constant. Varying brightness settings were only observed for survey flights 20200321_01_PS122-3_32-70, 20200423_02_PS122/3_37-66, 20200510_01_PS122/3_39-109 and 2020/07/04_01_PS122/4_45-112.

**Fig. 5:**
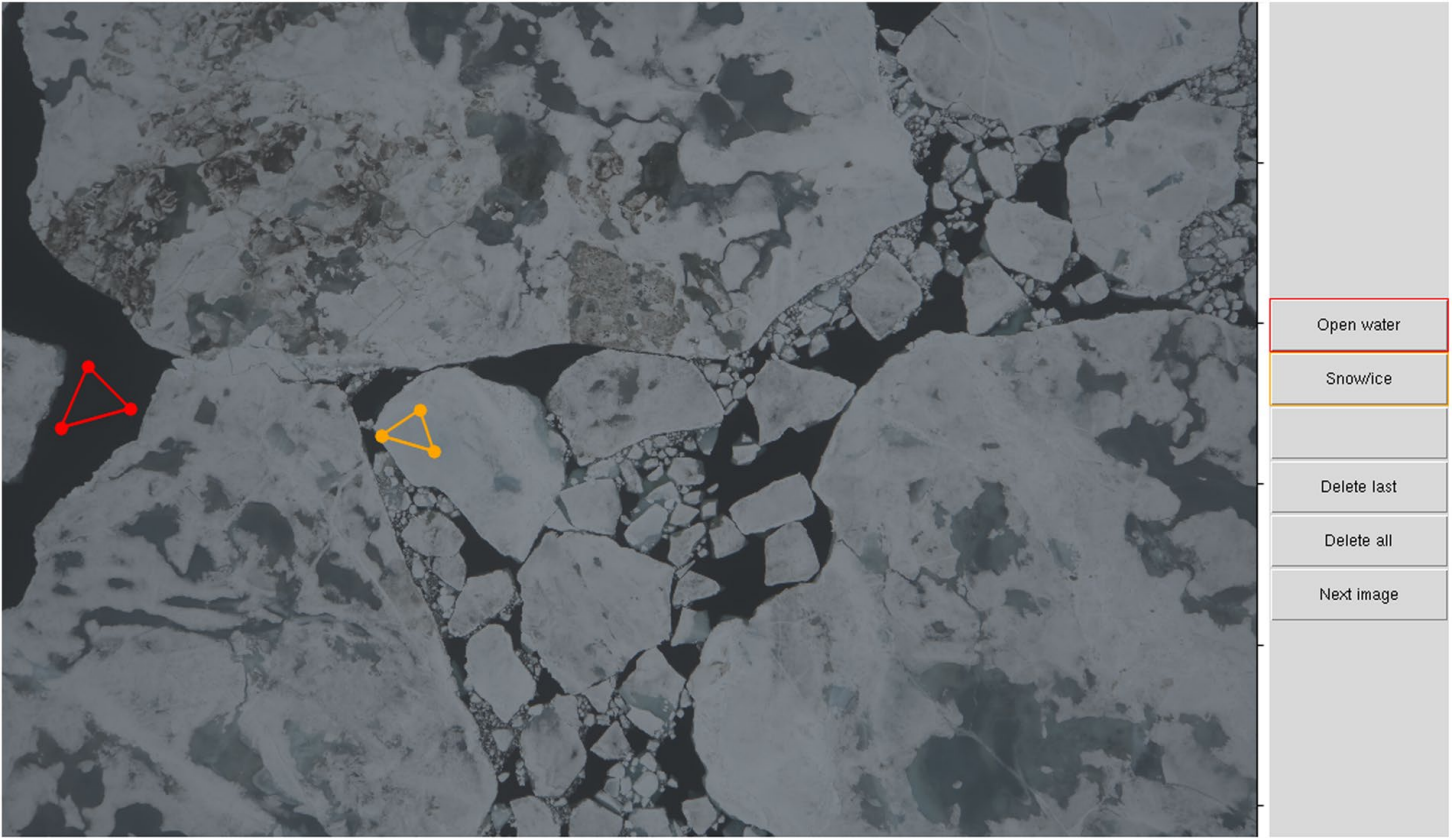
Graphical user interface to select ‘Open water’ areas and ‘Snow/ice’ covered areas. All images of the respective flights were linearly stretched between the selected minimum and maximum values respectively.

In the next step vignetting correction was applied to the TIFF images employing estimates from laboratory measurements at the outlet of an Ulbricht sphere following the calibration performed by Ehrlich *et al*.^12^. All TIFF images were converted into a lossless JPG file format being the best compromise between file size and quality^13^. In-flight GNSS (latitude, longitude, altitude) and INS (roll, pitch, yaw) measurements matching the second of image acquisition were assigned to each image. In order to use a unified classification independent of the image brightness, a second set of JPG images was created for each survey flight whose brightness was adjusted with an empirical line method. For this purpose, a graphical user interface has been implemented in which the operator is able to manually select flight-specific dark (open water) and bright areas (snow surfaces or bright areas in ridges after snow melt) from one representative image (Fig. 5). After selecting these minimum and maximum values all images of the flight were linearly stretched so that dark areas correspond to a reflectivity of 0.1 and light areas to 0.9, resulting in a set of brightness adjusted images of each survey flight (Fig. 3a). Both values were chosen as a compromise between making full use of the available data range and avoiding over- and undersaturation of pixels. This type of correction was chosen to improve the performance of subsequent image classifications. The chosen values thus do not reflect real reflectivities and therefore were not verified in more detail. Most of the conversion and pre-processing steps are part of a full classification suite specifically designed for helicopter aerial imagery^14^.

#### Initial drift correction

Compared to airborne surveys over static land areas sea-ice surveys can be influenced by the effects of sea-ice motion between data acquisitions. In the Arctic Ocean, sea-ice velocity peaks above 1 m s^−1^ with short-term variability primarily driven by the prevailing wind conditions^15–17^. During MOSAiC an average sea-ice velocity of 0.098 m s^−1^ (or 8.52 km d^−1^) has been reported by Krumpen *et al*.^18^ resulting in an average sea-ice displacement of ~700 m during a 2-hour helicopter flight. Therefore it is essential to correct the image locations for the effect of sea-ice motion prior to the SfM pipeline. Following a similar approach as Hyun *et al*.^8^ we employed the position of RV *Polarstern* at the time of image acquisition (Fig. 3b). For this, we used 1-minute RV *Polarstern* GNSS logs, available via the DSHIP database^19^, linearly interpolated to 1 Hz resolution. As a reference location, we used the geographic position of an image acquired at the approximate center time of the respective survey flight (Table 1). We then calculated the difference in meters between the position of RV *Polarstern* at the reference time and at the acquisition time of each image. Finally, these differences were used to roughly correct the image locations for the effects of sea-ice drift. Additionally, we corrected the INS-measured yaw of the helicopter by the angle difference of the GNSS-derived bearing before and after the drift correction. These drift-corrected parameters are employed in the SfM pipeline described in the next section in which the exact recording positions were finally reconstructed. A more precise initial drift correction was therefore not necessary. One of two exceptions from the initial drift correction is exploratory flight 20200616_01_PS122-4_44-78 where RV *Polarstern* was not moored to the CO ice floe but was still on its way back from Svalbard where the leg 3 to leg 4 crew exchange took place. To correct this specific flight for the effects of sea-ice drift we used 1 Hz GNSS logs from a meteorological surface flux sled^20^ which remained at the MOSAiC site during RV *Polarstern’s* excursion to Svalbard. The second exception is survey flight 20200921_01_PS122-5_63-3 where a highly non-linear drift pattern was observed in the second half of the survey. Therefore, no drift correction was applied to this specific survey as it would deteriorate the derived data products. Both exceptions are highlighted in Table 2.

**Table 2:** Overview of the resulting orthomosaics and DEMs.

Date	#Flight	DSHIP ID	RMSE_*no_drift*_ (m)	RMSE_*drift*_ (m)	DEM quality	Orthomosaic quality	Full res. (m)	STD_*ALS*_ (m)
2020-03-18	1	PS122/3_32-42	16.4	15.1	Poor	Good	0.16	0.52
2020-03-21	1	PS122/3_32-70	473.9	461.2	Moderate	Good	0.16	0.81
2020-03-23	1	PS122/3_33-17	461.2	87.8	—	Good	0.5	—
2020-04-23	1	PS122/3_37-63	105.8	54.8	Good	Good	0.15	0.11
2020-04-23	2	PS122/3_37-66	56.1	37.6	—	Good	0.5	—
2020-05-10	1	PS122/3_39-109	380.2	43.6	Good	Good	0.15	0.15
2020-06-16*	1	PS122/4_44-78	349.9	39.2	Poor	Moderate	0.5	1.59
2020-06-30	1	PS122/4_45-36	74.6	41.1	Good	Good	0.16	0.24
2020-06-30	2	PS122/4_45-37	63.4	31.5	—	Good	0.5	—
2020-07-04	1	PS122/4_45-112	197.3	14.7	Moderate	Good	0.03	1.2
2020-07-07	1	PS122/4_46-39	364.9	25.3	Moderate	Good	0.03	0.46
2020-07-07	3	PS122/4_46-39	550.9	47.8	—	Moderate	0.5	—
2020-07-11	1	PS122/4_46-97	179.9	21.6	—	Moderate	0.5	—
2020-07-17	1	PS122/4_47-96	198.4	26.6	Good	Good	0.4	0.71
2020-07-22	1	PS122/4_48-69	294.0	58.3	Moderate	Good	0.16	0.79
2020-08-06	1	PS122/4_50-32	152.1	89.7	—	Good	0.5	—
2020-08-07	1	PS122/4_50-45	91.3	12.9	—	Good	0.5	—
2020-08-18	2	PS122/5_59-139	39.5	15.1	—	Good	0.5	—
2020-09-07	1	PS122/5_61-62	225.5	18.3	Moderate	Moderate	0.03	1.12
2020-09-08	2	PS122/5_61-63	163.7	30.2	—	Moderate	0.5	—
2020-09-11	1	PS122/5_61-190	135.6	10.9	Good	Good	0.03	0.73
2020-09-15	1	PS122/5_62-67	156.2	25.5	Moderate	Good	0.03	0.79
2020-09-19	1	PS122/5_62-166	237.9	40.6	Poor	Good	0.15	0.56
2020-09-21**	1	PS122/5_63-3	30.8	30.8	—	Moderate	0.5	—

Next to the date, flight number and DSHIP^19^ ID, we list the RMSE before (RMSE_*no_drift*_) and after the drift correction (RMSE_*drift*_), the quality flags *Poor*, *Moderate* and *Good* for both the DEMs and the orthomosaics and the full resolution of the latter. Note that all DEMs and transect orthomosaics are gridded to a final resolution of 0.5 m while for the central part of the CO floe grid survey orthomosaics are available at full resolution. STD_ALS_ is the standard deviation of *photogrammetric* DEM - ALS DEM.

*GNSS logs from meteorological surface flux sled^20^ applied for drift correction.

**No drift correction applied.

#### Structure from motion pipeline

For calculating three-dimensional (3D) surface models of the ice floes surrounding RV *Polarstern* we employed an SfM pipeline on the drift-corrected images. A prerequisite of SfM to work is that each 3D feature is illuminated at least from two different viewpoints. Therefore a large image overlap is needed to obtain optimal results. Here we employed the SfM pipeline implemented in the Agisoft Metashape software^21^. To guarantee consistency of the single processing steps for each flight we employed Agisoft’s python programming interface instead of manual work within the graphical user interface of the software. This way we implemented a processing scheme that aligns the images of each survey flight in the first step, guided by the drift-corrected metadata and a pre-calibrated camera model (Fig. 3c). The latter is originating from a helicopter survey with the same camera setup as flown during the MOSAiC campaign but conducted on 2019-08-14 over land (i.e., fixed terrain) in northern Germany. However, due to different environmental conditions such as temperature and humidity this camera model only serves as an initial guess and is individually adapted for each flight during the initial image alignment. For the initial image alignment, we used the original images without any brightness corrections as recommended by Agisoft^21^ resulting in a sparse cloud of tie points. Initial intrinsic and extrinsic camera parameters and triangulated tie point coordinates were refined in the following camera optimization step employing a bundle adjustment procedure. Here the term ‘bundle adjustment’ refers to the optimization of light rays leaving each 3D feature with respect to both feature and camera positions^22^. In order to suppress noise in the point cloud, tie points with a high triangulation uncertainty were removed using Agisoft’s reconstruction uncertainty filter followed by a final camera optimization step. Following the standardized Agisoft workflow depth maps and a dense point cloud were built in the next step. At this step, the user is able to let Agisoft calculate confidence values for each point in the dense point cloud which can be used later to remove erroneous measurements. This confidence value is defined as the number of contributing combined depth maps per point^21^. We, therefore, decided not to use it for filtering as this would remove large parts of the final orthomosaics in regions with minimal image overlap. We instead provide a confidence grid that can be employed by the user to identify low confidence grid points in the final data products (Figs. 3d and 8d,h,l).

#### Filtering of dense point cloud

After exporting the dense point cloud and the associated confidence values from Agisoft Metashape we employed the open3D library for spatial filtering^23^. In the first step, we used the implemented RANSAC algorithm to fit the best plane through the dense point cloud (Fig. 3d). All points with an offset in altitude of >50 m from the plane were removed from the point cloud. This threshold seems reasonable as we presume RV *Polarstern* is the highest resolvable obstacle in the wider region having a keel to maximum chimney height of 51.45 m and a draft of 11.21 m^24^. The remaining point cloud was further filtered by a statistical outlier removal which removes points that are further away from their neighbors when compared to the average of the point cloud^23^. The filtered point cloud was then gridded to 0.5 m resolution resulting in a DEM of the ice floes and an associated confidence map (Fig. 3d).

#### Gridding and projection of orthomosaics

**Fig. 6:**
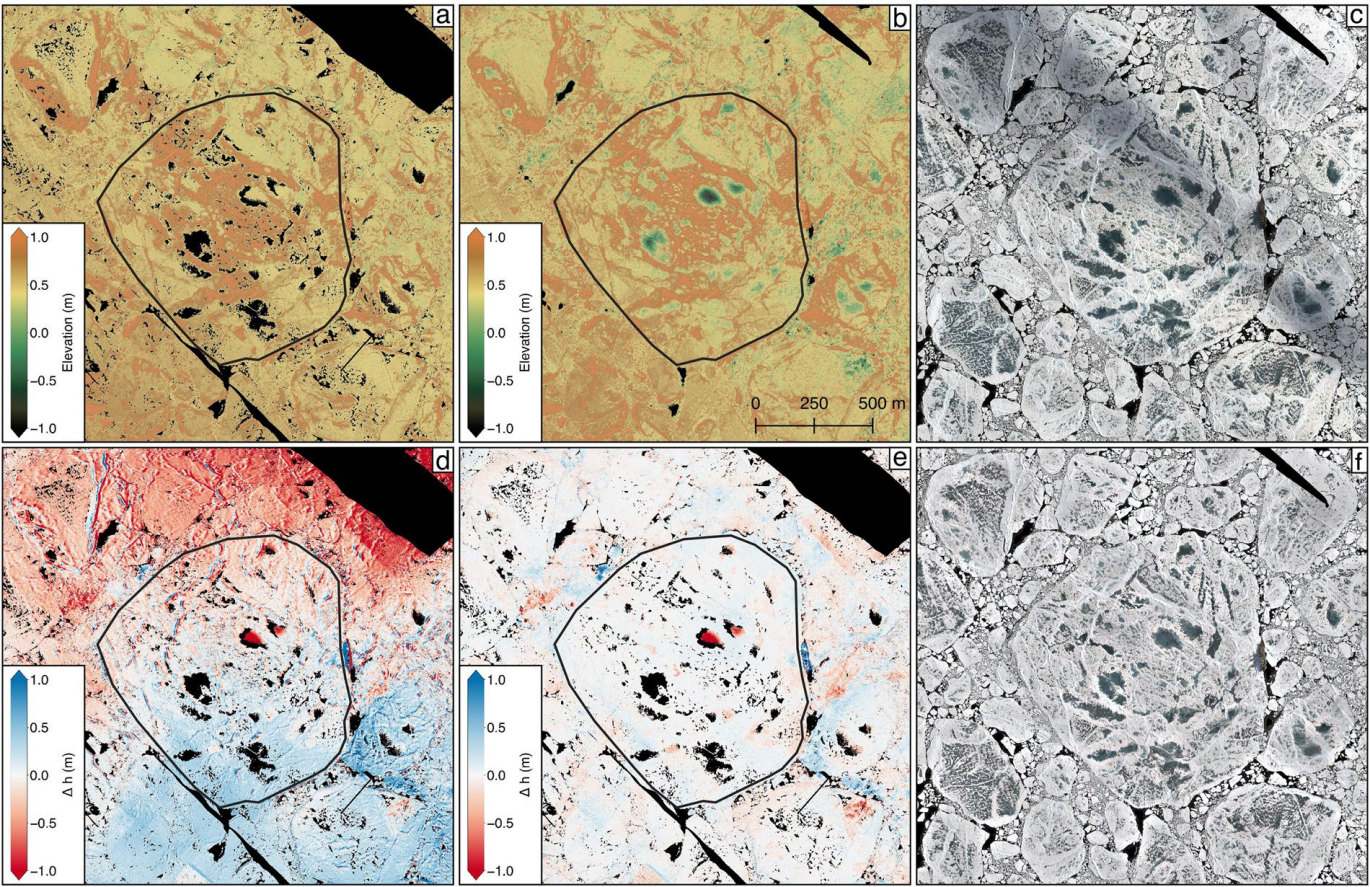
Combination of photogrammetric and ALS datasets of survey flight 20200630_01_PS122-4_45-36. ALS surface elevation measurements (**a**) and spatially adjusted photogrammetric elevation estimates (**b**) are shown next to the original orthomosaic (**c**), Δ*h* = original photogrammetric DEM - ALS DEM (**d**), Δ*h* = adjusted photogrammetric DEM - ALS DEM (**e**) and the orthomosaic corrected for cloud shadows employing ALS reflectance measurements (**f**). The leg 4 Central Observatory (CO) ice floe is framed by the black line.

To produce the final orthomosaic datasets the filtered dense point cloud was re-imported into Agisoft Metashape. Based on this a temporary DEM was built internally including an interpolation step of small data gaps (Fig. 3e). This step is necessary to avoid no data voids (i.e. over open water) in the final orthomosaics. All images were projected on the void-filled DEM and seamless orthomosaics were generated. Here it should be noted, that the final data product includes the DEM with data voids (e.g. Figure 6b).

Next to the orthomosaics of the original images we also created orthomosaics for the brightness adjusted datasets (Fig. 3e). The final brightness corrected orthomosaics were gridded to a ground resolution of 0.5 m. For grid flights covering the position of RV *Polarstern*, we also extracted the region of the CO ice floe at full spatial resolution. The latter largely depends on the respective flight altitude of the helicopter and varies between 0.03 and 0.5 m. All datasets were projected to a polar stereographic reference system centered at 45° W (EPSG:3413) and vertically referenced to the DTU21 Mean Sea Surface grid provided by DTU Space^25^. Finally, it should be noted that due to the minimal image overlap in the along-track direction and no side overlap within the L-site triangle flights, butterfly surveys, and transect flights only coarse surface reconstructions were possible for these surveys. Therefore, we only provide DEM products for the CO grid flights.

### Combination of camera and ALS data

During all camera surveys a Riegl VQ580 ALS was routinely employed to map the topography of the ice floes surrounding the wider area of RV *Polarstern* (Fig. 1a,b). In order to compare and adjust the photogrammetric DEMs obtained in this study we used contemporaneous acquired ALS surface height and reflectance measurements. Here we used gridded data products available at a spatial resolution of 0.5 m^26^. An example of the ALS surface height product of CO floe grid flight 20200630_01_PS122-4_45-36 is shown in Fig. 6a. While ALS data from grid surveys were corrected for the effects of sea-ice drift employing a similar strategy as applied in this study, no such drift correction was applied to ALS data from transect and triangle flights.

In order to co-register the derived photogrammetric DEMs to the respective ALS surface elevation measurements we employed the pc_align utility provided within NASA’s Ames Stereo Pipeline^27^. Here we used an alignment procedure that searches two input datasets for matching features. For this, hillshaded versions of the input DEMs were produced by creating a hypothetical illumination of the DEMs. Among these hillshaded DEMs point matches were calculated using an iterative closest point algorithm resulting in a 4 × 4 matrix including information on the translation, rotation, and scaling between both datasets^27,28^. Finally, this information was applied to the photogrammetric DEMs to match the ALS grids. In the last step, we subtracted a smoothed version of the ALS DEMs to remove large-scale vertical biases from the aligned photogrammetric DEMs. For smoothing the ALS DEMs we tested several kernel sizes and found the average within a 250 m × 250 m moving window appropriate for our needs. Figure 6d shows the differences between the original photogrammetric DEM and the ALS DEM acquired during survey flight 20200630_01_PS122-4_45-36 (Fig. 6a). Here biases in both, horizontal and vertical directions become evident between both datasets. Figure 6e shows the same differences but after the horizontal alignment and vertical bias removal described above. An example of the final photogrammetric DEM is shown in Fig. 6b. Next to the photogrammetric DEMs we shifted the corresponding orthomosaics employing the derived matrices from the feature-based alignment procedure resulting in a co-registered stack of RGB orthomosaic data and ALS surface elevation and reflectance measurements. This data stack is a prerequisite to compensate for cloud shadows in the orthomosaic data as described in the next paragraph.

Some of the derived orthomosaics are clearly disturbed by shadows originating from clouds situated above the helicopter during data acquisition (e.g., Fig. 6c). Different to passive image capture, active ALS measurements are not influenced by high-level clouds^29^. We, therefore, hypothesize that orthomosaic intensity values of shaded areas are relatively low when compared to any cloud-free neighboring areas and $R,G,B < AL{S}_{refl}$ should hold true in shaded areas. Here *ALS*_*refl*_ corresponds to gridded and normalized ALS reflectance values and *R, G, B* corresponds to the single bands of the aligned orthomosaics. Under this assumption we calculated a scaling factor (*sf*) for each pixel and band (*R, G, B*) by

2 $$sf=\frac{AL{S}_{refl}}{R,G,B}$$

resulting in *sf* > 1 for shaded areas and *sf* < 1 in areas not influenced by cloud shadows. Here it should be noted that both *ALS*_*refl*_ and *R, G, B* were filtered by a Gaussian smoothing operator with a 250 m kernel size beforehand in order to suppress noise and to extrapolate the narrower swath width of the ALS dataset. For areas not influenced by cloud shadows, i.e., where *sf* < 1 we included a threshold at *sf* = 0.65 to keep the unaffected intensity values at a reliable level. By multiplying the single bands of the original orthomosaics with their individual *sf* maps we obtained a clear improvement in areas influenced by cloud shadows (Fig. 6f).

## Data Records

The orthomosaic and DEM data derived in this study are available at 10.1594/PANGAEA.949433^30^. CO grid flights are available as single grids while transect and triangle flights were segmented into 2 km × 2 km data tiles to provide the user with manageable file sizes. Next to the orthomosaic and DEM data we provide confidence maps of the respective survey flights. All data are stored in GeoTIFF file format and gridded to 0.5 m spatial resolution. For the CO grid flights, we also provide orthomosaic data at full spatial resolution within a 3 km square centered on RV *Polarstern*. The naming convention of the final data products is: *Date, #Flight, DSHIP ID* followed by *DEM*, *confidence* or *orthomosaic* and *hr* for high resolution (0.5 m) or *fr* for full resolution (Table 2). As we provide brightness-corrected orthomosaics these are termed *l2* for level 2 products and if the data were corrected for the effect of cloud shadows we added a *l2b* product. All final datasets are stored in one zip archive per survey flight.

## Technical Validation

**Figure 7:**
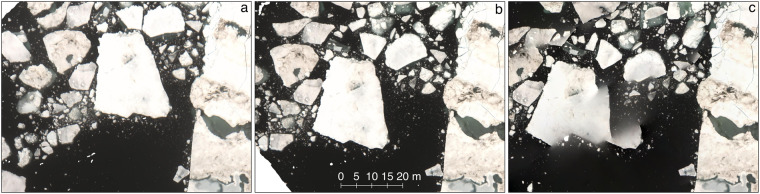
Orthorectified images of survey flight 20200717_01_PS122-4_47-96 with data acquired at 15:43:09 (**a**) and 15:48:41 (**b**) are shown next to the resulting orthomosaic (**c**). Here it becomes evident that the smaller ice floes on the left side of the images are drifting at another speed than the larger ice floe on the right side of the images. This discrepancy can not be compensated by the applied image blending (**c**) and remains an issue for orthomosaic data of smaller ice floes.

By applying the processing scheme described above we generated 24 orthomosaics covering legs 3 to 5 of the MOSAiC expedition (Fig. 1c). It is important to note that the accuracy of each orthomosacic is a function of the underlying images, including image overlap (itself a function of the flight pattern), illumination, camera settings, as well as prevailing sea-ice concentration. Next to the derived confidence maps we, therefore, introduce the qualitative flags *poor*, *moderate* and *good* which are based on visual inspections of the datasets to provide the user with an additional reliability measure of the derived DEM and orthomosaic products (Table 2). For the DEMs, these qualitative flags are built on (1) number of contributing depthmaps, (2) DEM detail, and (3) obvious data jumps while the orthomosaics were investigated for obvious interpolation artifacts (e.g. Fig. 7) and data jumps. We further investigated the impact of the applied drift correction by comparing the Root-Mean-Square Errors (RMSE) of the drift-corrected camera positions to the original camera positions. For this we calculated the total RMSE (Table 2, *RMSE*_*no_drift*_) between the initial camera positions and the estimated camera positions from the bundle adjustment of the SfM pipeline by

3 $$RMSE=\sqrt{\frac{{\sum }_{i=1}^{n}{\left({X}_{iref}-{X}_{iest}\right)}^{2}+{\left({Y}_{iref}-{Y}_{iest}\right)}^{2}+{\left({Z}_{iref}-{Z}_{iest}\right)}^{2}}{n}}$$

where *X*_*iref*_, *Y*_*iref*_ and *Z*_*iref*_ are the X, Y and Z coordinates of the camera as measured by GNSS and *X*_*iest*_, *Y*_*iest*_ and *Z*_*iest*_ are the X, Y and Z coordinates of the camera as estimated from the bundle adjustment. We also calculated the RMSE between the drift-corrected camera positions and the camera positions from the respective bundle adjustment (Table 2, *RMSE*_*drift*_). Both RMSE values are shown in Table 2 and summarize the impact of the applied drift correction. By applying an initial drift correction to the images the RMSE decreased between 3 to 92% for all flights (except survey flight 20200921_01_PS122-5_63-3). The remaining error can be attributed to a combination of differential sea-ice motion in the survey area, time shifts between image acquisitions and GNSS logs and inaccurate INS measurements. As evident from Table 2 the lowest RMSE improvement is found for survey flight 20200321_01_PS122-3_32-70. As it is also the longest flight investigated in this study (Table 1) we attribute this to differential sea-ice motion in the survey area during data acquisition.

Interpolation errors in the orthomosaic data also occur when smaller ice floes (meter scale size) drift at different speeds than nearby larger ice floes. An example is shown in Fig. 7. Here we show two orthorectified images acquired during survey flight 20200717_01_PS122-4_47-96 (Fig. 7a,b) and the resulting orthomosaic (Fig. 7c). Consistency exists in the right part of the images where a larger ice floe is drifting at a constant speed in between data acquisitions. However, in the left part of the images smaller ice floes are drifting at a different speed and in different directions (Fig. 7a,b) resulting in artefacts in the final orthomosaic data (Fig. 7c). As these interpolation errors are restricted to the occurrence of smaller ice floes within lower sea-ice concentration, these issues are only observed for leg 4 data acquired between June and August 2020 (Table 1).

**Figure 8:**
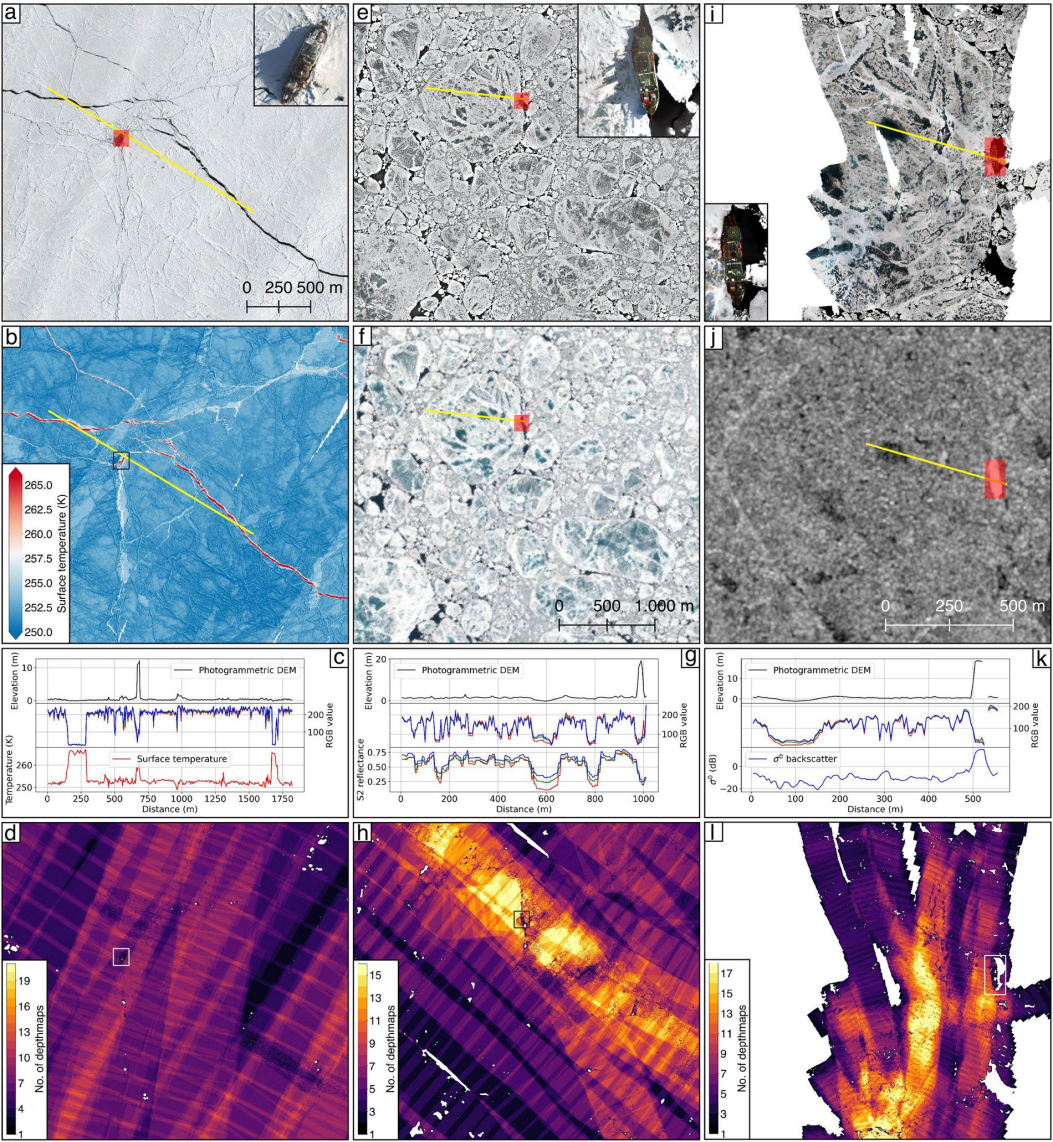
Comparison of example orthomosaics to independent reference data. Orthomosaic data of survey flight 20200423_01_PS122-3_37-66 (**a**) is compared to a map of contemporaneous acquired sea-ice surface temperature data^31^ (**b**). The profile indicated by the yellow line is shown in (**c**) including photogrammetric DEM, orthomosaic, and surface temperature data^31^. The corresponding confidence map is shown in (**d**) Orthomosaic data of survey flight 20200630_01_PS122-4_45-36 (**e**) is compared to a Sentinel-2 scene acquired 06:04:07 (HH:MM:SS) after the center time of the respective helicopter survey (**f**). Profile in (**g**) shows the same data types as in c but with Sentinel-2 reflections instead of surface temperature estimates. The corresponding confidence map is shown in (**h**) Orthomosaic data of survey flight 20200704_01_PS122-4_45-112 (**i**) is compared to a TerraSAR-X scene acquired 05:08:19 (HH:MM:SS) after the center time of the respective helicopter survey (**j**). Profile in (**k**) includes photogrammetric DEM, orthomosaic and TerraSAR-X backscatter (*σ*^0^) data. The corresponding confidence map is shown in (**l**) Location of RV *Polarstern* is highlighted for all dates at a scale of 1:5000.

We further compare some orthomosaic data to temporally close reference observations if available. In particular, we rely on Sentinel-2 and TerraSAR-X satellite data and a helicopter-borne sea-ice surface temperature map acquired by an infrared camera during survey flight 20200423_01_PS122-3_37-66 (Fig. 8). The latter is based on thermal infrared brightness temperatures converted into a map of surface temperatures at 1 m spatial resolution^31^. Sentinel-2 data were downloaded via the Google Earth Engine and are available at 10 m spatial resolution^32^. For comparison we used an RGB composite of band-4, band-3 and band-2 of the Sentinel-2A satellite with data acquired on 2020-06-30 (Fig. 8d). The Sentinel-2A scene was acquired 06:04:07 (HH:MM:SS) after *t*_*center*_ of helicopter survey 20200630_01_PS122-4_45-36. On 2020-07-04 a TerraSAR-X scene was acquired in stripmap mode over the leg 4 ice floe. We employed the Multi Look Ground Range Detected data product provided by the German aerospace centre (DLR). The TerraSAR-X scene was reprocessed to a geocoded *σ*^0^ backscatter map at 3.5 m spatial resolution employing the GAMMA remote sensing software^33^ (Fig. 8f) and was acquired 05:08:19 (HH:MM:SS) after *t*_*center*_ of helicopter survey 20200704_01_PS122-4_45-112. When comparing the orthomosaic data to these reference datasets it should be noted, that we did not apply any additional scaling to the orthomosaic data to match the reference datasets but just used a simple translation and rotation to compensate for the sea-ice drift in between data acquisitions. When investigating the profiles in Fig. 8c,g,h all orthomosaics agree within the pixel spacing of the coarser reference datasets. Furthermore we measured the length of RV *Polarstern* in the final orthomosaics and find values of 118 ± 1 m which matches the technical specification of the vessel^24^. When concerning the dynamic sea-ice environment surrounding the static vessel, this length scale is considered a rather simple but robust measure of confidence.

As the derived DEMs of the CO grid surveys were horizontally and vertically adjusted to the ALS surface elevation maps these cannot be used to validate the photogrammetric DEMs. However, some conclusions can still be drawn from the standard deviation of Δ*h* = *photogrammetricDEM-ALSDEM* (STD_*ALS*_) as shown in Table 2. (1) a low STD_*ALS*_ indicates a good horizontal and vertical alignment of both datasets. (2) A high STD_*ALS*_ indicates outliers in one of the datasets which can be attributed to processing artifacts or to the different methods used for data acquisition. When investigating Fig. 6a,b largest differences are evident in the areas of melt ponds. As the employed Riegl VQ580 ALS is operating in the near-infrared spectrum, most of the laser signal is absorbed by water resulting in a weak backscatter compared to the surrounding sea-ice surface. This backscatter originates mostly from the water surface due to the minimal penetration depth of the near-infrared laser beams^34^. In the visible spectrum of the camera, more light is transmitted through the water until it reaches the floor of the melt pond. Neglecting the refraction of water for a moment, the photogrammetric DEM, therefore, represents the melt pond bathymetry while the ALS DEM represents the surface of the ponds resulting in the pronounced elevation differences mentioned above.

## Usage Notes

The derived orthomosaics, DEMs and confidence maps are available in GeoTIFF format and can readily be imported into any Geographical Information System such as ArcGIS or QGIS for visualisation. We used a standardized polar stereographic coordinate system provided by the National Snow and Ice Data Center (NSIDC), available at EPSG code 3413. The derived orthomosaic data may be useful for scientists working on *in situ* snow and albedo measurements to extrapolate their findings to the wider area of the respective CO ice floe. Full resolution orthomosaics might also become handy for identifying Remotely Operated Vehicle (ROV) surveys from underneath the sea ice using identifiable features on the surface. As already shown in Fig. 8 the derived orthomosaic data can also be compared to temporally close satellite observations in order to validate surface classification results of the latter. For this, the orthomosaic data can be classified into different surface types employing publicly available classification schemes^14,35^. High resolution classification results can give important insights into the spatial distribution and geometric properties of melt ponds during the drift of RV *Polarstern*. Here it should be noted that in the case of cloud cover shaded areas might be falsely classified as melt ponds. Therefore, we suggest to use the shadow-corrected level 2b product for any classification purposes. In addition, to melt pond retrievals, classification results of larger survey flights can also be used to map lead fractions and floe size distribution. Furthermore, differences in melt pond and ocean color might be applicable for indicating different algae species.

In addition to the optical orthomosaic data we provide the user with photogrammetrically derived DEM products of the sea ice surrounding RV *Polarstern*. As evident from Table 2 these are of different quality, which is dependent on the number of depth maps contributing to each DEM pixel (Fig. 8d,h,l). The DEM and orthomosaic products can therefore be investigated by the user employing the provided confidence maps available for each survey flight. These can also be used to mask out low-confidence regions in the DEM and orthomosaic datasets. When investigating the differences between the photogrammetric DEMs and the ALS derived surface measurements (e.g. Fig. 6e) it becomes evident that the largest differences occur in areas of melt ponds. While the ALS measures the laser pulse returns from the water surface, visible radiation is able to penetrate clear melt pond water. After correcting the images or point cloud for the refraction of water this capability has already been used to map coastal bathymetry elsewhere^4,36^ and photogrammetric DEMs might assist the evaluation of melt pond depth and volumes on sea ice.

## Data Availability

All processing developed within this study is wrapped in a python environment and is available at https://gitlab.com/mosaic12/orthomosaics. For calculating image footprint locations we used the *CameraTransform* python package^37^. Note that part of the code is based on the commercial Agisoft Metashape software requiring licensing.
